# Leptin Methylation and mRNA Expression Associated With Psychopathology in Schizophrenia Inpatients

**DOI:** 10.3389/fpsyt.2022.793910

**Published:** 2022-02-07

**Authors:** Jiaqi Song, Yan Chen, Qing Zhao, Hongna Li, Wei Li, Ke Chen, Jianjin Yu, Weihong Fu, Dachun Chen

**Affiliations:** ^1^Huilongguan Clinical Medical School, Beijing Huilongguan Hospital, Peking University, Beijing, China; ^2^Beijing HuiLongGuan Hospital, Beijing, China

**Keywords:** DNA methylation, leptin, mRNA, expression, schizophrenia

## Abstract

Leptin involved in the regulation of dopaminergic neurons of the central nervous system may confirm the hypothesis of neurodevelopment in schizophrenic patients. However, specific genetic mechanisms are undefined. Therefore, we aimed to explore the regulation of DNA methylation of leptin promoters and mRNA expression in patients with schizophrenia. A cross-sectional study enrolled 40 patients and 40 healthy controls from the Beijing Huilongguan Hospital in China. The leptin methylation levels and mRNA expression were examined by highly sensitive mass spectrometry based on the MassARRAY System and real-time quantitative polymerase chain reaction (qPCR). The Positive and Negative Symptoms Scale (PANSS) was applied to estimate the clinical symptoms of patients. The *LEP*-CpG7 and CpG15 methylation in patients were significantly higher than in healthy controls (*P* < 0.05). The *LEP*-CpG11, CpG33.34.35, CpG36 methylation, and mRNA expression decreased significantly in patients compared with healthy controls (*P* < 0.05). After controlling gender, age, BMI, dose of antipsychotic and duration of illness, *LEP*-CpG7 methylation was negatively associated with PANSS positive symptoms subscore (*r* = −0.485, *P* = 0.005). In addition, *LEP*-mRNA expression was negatively correlated with PANSS total score (*r* = −0.385, *P* = 0.03) and positive subscale (*r* = −0.392, *P* = 0.026). Nevertheless, only the *LEP*-CpG7 methylation level remained negatively correlated to the PANSS positive subscore following multiple stepwise regression (β = −17.071, *P* = 0.037). These results suggest that leptin methylation and mRNA expression might contribute to the pathogenesis of schizophrenia. *LEP*-CpG7 methylation may be negatively associated with positive symptoms in patients with schizophrenia.

## Introduction

Schizophrenia is deemed to a chronic and severe psychosocial disease with a lifetime prevalence of ~1% of the world's population (1), which has a profound effect on individuals, families, and society. Moreover, a fraction of them have chronic symptoms, cognitive impairment and mental disability (2). However, the exact cause remains unknown. There are many theoretical hypotheses about neurotransmitters, neurodevelopmental, inflammatory response, and so on (3). Evidence of various genetic defects, such as gene polymorphisms, mutations, copy number variants, and translocation in schizophrenia (4) explains only small portion of cases. Recent research has formulated more evidence for genetic heritability as well as critical interactions (epigenetic) between “hot” candidate genes and the environment for psychopathology of patients (5, 6). DNA methylation, one of the most widely associated epigenetic mechanisms, may has contributed to schizophrenia, either as a major etiological factor or as a target of antipsychotic epigenetics (6).

DNA methylation primarily occurs on cytosine residues in CpG dinucleotides close to gene promoters. The process is catalyzed by DNA methyltransferases, without changing the gene sequence (7). It has been shown that DNA methylation induces or inhibit genes mRNA expression after facilitating or blocking accessibility of transcription factors to the of gene promoters (8). DNA methylation of cytosines in CpG sites has proved sensitive to environmental stimuli (9, 10). By contrast, genotypes themselves are relatively inherently stable during duplication. This might be why one of monozygotic twins later becomes a schizophrenic patient, while the other is well. Several previous studies reported that aberrant DNA methylation and mRNA expression of neurodevelopmental genes, such as RELN, BDNF (11) etc. might contribute to schizophrenia pathogenesis and be associated with its clinical manifestation (12, 13).

Leptin, a 16 kD protein, a primary product of the Ob gene, is an abundant hormone synthesized and secreted by adipocytes in adipose tissue, consistent with the amount in peripheral blood (14). Additionally, evidence shows that leptin is secreted by the human brain and pituitary gland with an abundance of leptin receptors (15). Leptin was shown to maintain the body energy balance, and plays a documented role in regulating brain development, reproductive system, immune system, and bone development (16). Leptin sends an important signal to the mesolimbic dopamine system with stimuli to the lateral hypothalamic area, to regulate dopaminergic and serotonergic neurotransmitter systems and brain crucial regions related to behavior and emotion regulation (17). It is well known that enhanced activity of the dopamine system in the limbic-midbrain area has been implicated in the pathogenesis of mental disease, including schizophrenia (18).

Recently, the hypothesis of leptin dysregulation has been associated with psychopathology in schizophrenia. A case-control study illustrated that lower leptin levels in peripheral blood was closely related to more severe positive symptoms of schizophrenia (18). In contrast, another study revealed that higher levels of leptin in schizophrenic patients taking antipsychotic treatment, than healthy groups (19). However, it is still unclear how leptin dysregulation works in the schizophrenic process. More results propose that DNA methylation seems to explain the problems caused by inherited and environmental interactions. To date, there is no relative report exploring the potential pathogenesis and psychopathology of schizophrenia regarding DNA methylation regulation and mRNA expression of leptin. Hence analyzing leptin CpG sites methylation and *LEP-*mRNA expression in peripheral blood are utile and profound means of exploring the mechanisms of action of leptin that underlie complex variation in mental and psychological features of schizophrenia.

Based on the studies described above, the aims of the current study were as follows:

1. To detect dysregulation of leptin CpG site methylation in the promoter region and mRNA expression in peripheral blood leukocytes, by comparing schizophrenia and healthy controls (HC) using the MassARRAY System and RT-PCR.
2. To explore the relationship between leptin methylation, mRNA expression and the psychopathological status of schizophrenia patients as assessed by the Positive and Negative Symptom Scale (PANSS).
3. To evaluate potential clinical and biological indices that could predict the severity of psychiatric symptoms in schizophrenia.

## Methods

### Subjects

All patients were recruited as inpatients of Beijing Huilongguan Hospital, China, and HC were recruited via written and electronic advertisements in the local community from October 2017 to March 2018. This study initially enrolled 49 Chinese Han schizophrenic patients and 42 healthy subjects, who were matched for gender, age, and BMI.

All patients with schizophrenia was rigorously diagnosed by the fourth edition of the Diagnostic and Statistical Manual of Mental Disorders (DSM-IV). The inclusion criteria for patients were as follows: (1) between 18 and 60 years old; (2) right-handed; (3) on stable antipsychotic treatment for longer than 6 months (without dose change for at least 4 weeks). The daily chlorpromazine equivalent dosages were shown in Table 1. The exclusion criteria for inpatients were following: (1) were diagnosed with schizoaffective disorder, intellectual disability, substance abuse, mental disorder caused by organic brain disease; (2) had a severe endocrine, fever, immune and metabolic diseases, such as diabetes, other than those related to antipsychotics; and (3) current pregnancy or lactation.

**Table 1:** Demographics and clinical assessment of schizophrenia and healthy controls.

	**Schizophrenia** **(*n* = 40)**	**Healthy controls** **(*n* = 40)**	***t* or χ^2^**	***P-*values**
Gender (male/female)	20/20	20/20	1.251	0.263
Age (years)	46.1 ± 8.01	42.95 ± 9.58	−1.596	0.115
Years of education (years) BMI (kg/m^2^)	10.88 ± 2.88 24.71 ± 4.93	12.45 ± 3.89 24.4 ± 2.60	2.057 −0.353	0.043^*^ 0.725
Duration of illness (months)	271.05 ± 117.92	NA		
Dose of antipsychotics (mg) (chlorpromazine equivalents) PANSS	359.07 ± 166.31	NA		
Positive subscale	17.53 ± 5.88	NA		
Negative subscale	24.68 ± 5.99	NA		
General psychopathology subscale	34.23 ± 6.44	NA		
Total score	78.12 ± 17.40	NA		
Depressed subscale	6.85 ± 2.80	NA		

*BMI, body mass index; PANSS, Positive and Negative Syndrome Scale. Depressed subscale was the total score of G2, G3 and G6 from the General psychopathology subscale. Data are presented as mean ± SD*.

* *P < 0.05*.

The inclusion criteria for HC were as follow: (1) between 18 and 60 years old; (2) were right-handed; (3) no history of medicine or neurological or psychiatric disorders. The exclusion criteria for the controls subjects were following: (1) a family history of mental disorders among first-degree relatives; (2) were pregnant or lactation; (3) an Intelligence Quotient (IQ) < 70; (4) healthy controls with the endocrine, fever, immune and metabolic disease were also excluded.

**Figure 1:**
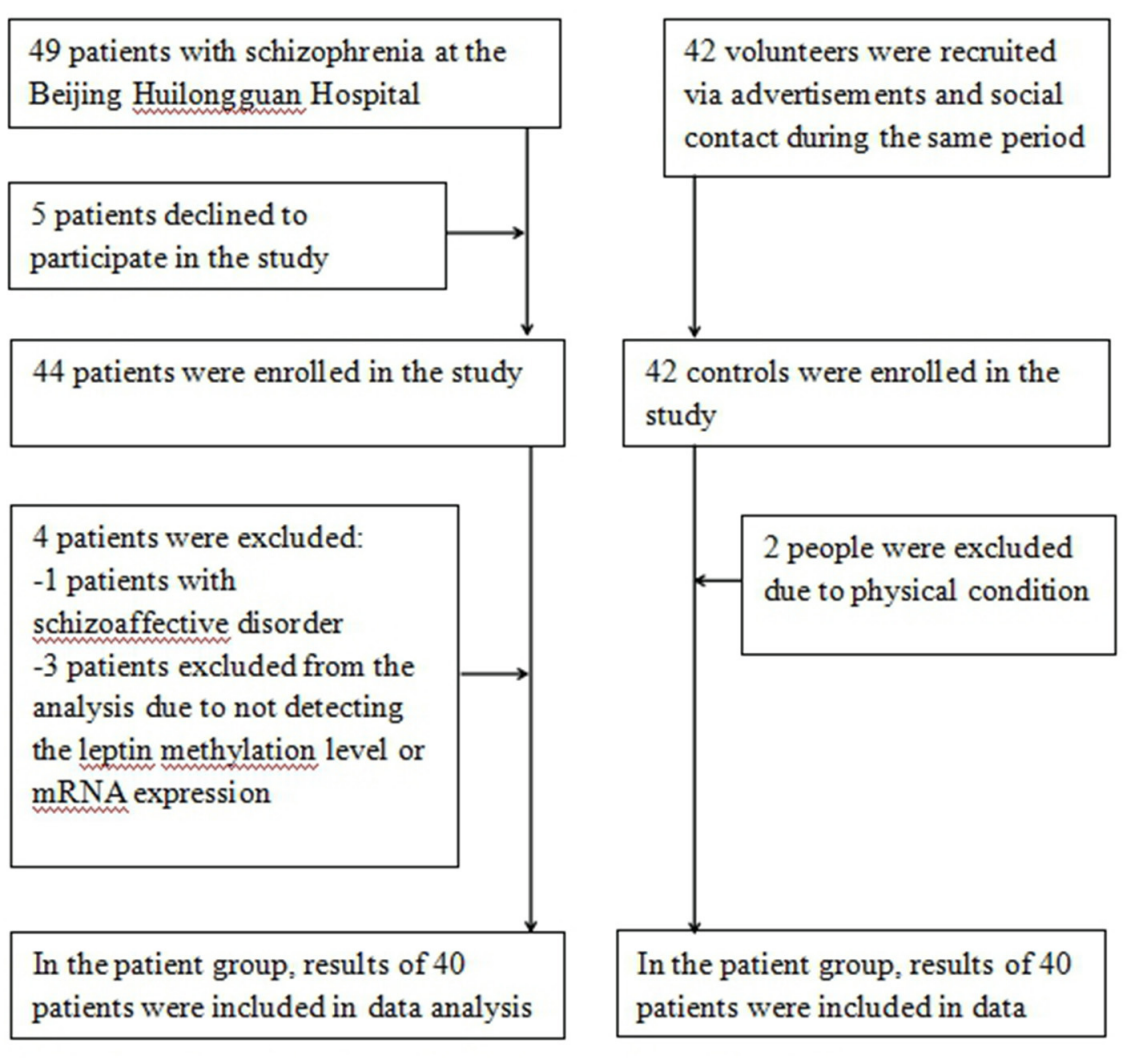
Overview of the flow of participants through the study.

Following screening, nine patients were excluded from the analysis due to not detecting the leptin methylation level or mRNA expression (three patients) or rejection (five patients) or exclusion criteria (one patient). They were therefore considered outliers. Forty HC were finally included in the analysis while two healthy people were excluded due to physical conditions. Hence, all analyses were performed on 40 inpatients and 40 controls in the study (Figure 1).

The study was approved by the Ethics Committee of Beijing Huilongguan Hospital and was consistent with the Declaration of Helsinki (version 2004). All participants, including schizophrenia and HC signed written informed consent after a detailed explanation of the study contents.

### Measures

#### Clinical Symptoms Assessments

The PANSS (20) was used to evaluate the severity of clinical symptoms of schizophrenic patients by two experienced psychiatrists to authenticate the accuracy of our data. Thereafter, an intraclass correlation coefficient (ICC ≥ 0.8) was maintained among repeated assessments. Antipsychotic dosages were replaced by chlorpromazine equivalents for each patient (21). Additionally, we also collected some patient information regarding duration of illness (months) and BMI (kg/m^2^), which was computed by height and weight.

In particular, depressed factor (total score of items G2, G3, G6 in the general psychopathology subscale), one of five factor models of PANSS was found to connect with clinical features and biomarkers in recent studies (22–24). Hence, we listed the depressed subscale separately for identical analysis.

#### Leptin DNA Methylation

During 6:00 and 8:00 am, fasting peripheral blood from HC and schizophrenia patients was collected in evacuated tubes with EDTA-K2, which aimed to extract DNA from blood leukocytes. Gene DNA was extracted from whole-blood samples using a Gentra Puregene Blood Kit (Qiagen, Germany) following the manufacturer's protocol. The quality of leptin DNA was assessed by OD values (1.5 < OD_260/280nm_ < 2, OD_260/230nm_ > 0.6) and gel electrophoresis detection (>20 ng/uL). DNA methylation levels at CpG sites in the promoter region were assessed using mass spectrometric technology, which proved to have high sensitivity, superior performance, high resolution, and easy repeatability on mass spectrometry based on the MassARRAY System (Sequenom, USA) for quantitative real-time sequencing of DNA methylation (25).

The primers and probe of leptin gene were designed accurately and comprehensively, which were based on two studies (26, 27). The primer for Leptin gene was developed with the Agena EpiDesigner (http://www.epidesigner.org) for CpG islands methylation prediction. The probe was designed according to the basic property requirement of Taqman probes. The primer sequences for human leptin gene were 5′-aggaagagagATTTAGAGTTGTGTGGGGTTTTGT-3′ (forward) and 5′-cagtaatacgactcactatagggagaaggctCACCTTCCCAAAAAACTAATCCTTA-3′ (reverse). *LEP* DNA methylation was measured at CpG sites near 222~648 bp in the promoter region of leptin. There was a portion of CpG sites without detection and some neighboring CpG islands to merge analysis. Finally, 12 CpG islands of leptin promoter were found to be involved in the analysis (Supplementary Figure 1).

#### Leptin mRNA Expression Measurements

Total mRNA collected in BD PAXgene Blood RNA tubes (Becton, Dickinson and Company, U.S.A) was extracted using the QIAamp RNA Blood Mini Kit (Qiagen Company, Germany). The quality of RNA was evaluated by its absorbance at OD_260/280nm_. Samples with a ratio above 1.8 (OD_260/280nm_ > 1.8) were adopted. Following the manufacturer's protocol, reverse transcription was induced by reverse transcriptase to cDNA. The primer sequences for leptin mRNA were 5'-AGCTGTGCCCATCCAAAAAG-3′(forward) and 5′-TGGAGGAGACTACTGCGTG-3′(reverse). Due to the relatively constant expression in blood, tissue, and organs, GAPDH (glyceraldehyde-3-phosphate dehydrogenase) was regarded as the reference gene when comparing leptin mRNA expression. The primer sequences for GAPDH were 5′-CTGCCAACGTGTCAGTGGTG-3′ (forward) and 5′-TCAGTGTAGCCCAGGATGCC-3′ (reverse). Real-time PCR (RT-PCR) was operated with a QuantiTect SYBR Green RT-PCR kit (Qiagen, USA). And in the end, the comparative cycle threshold (2^−ΔΔCt^) method was used to accurately calculate relative quantitative leptin mRNA expression with GAPDH as a housekeeping gene (28).

### Sample Size

This was the first exploratory study about the relationship between leptin methylation and schizophrenia. There was no reference to calculate sample size in previous studies. So we included 40 cases in each group according to the actual clinical situation and usable expenses, which was it know that this is incomplete for a rigorous clinical study. However, we believe that the sample size will be accurately calculated according to this study in our follow-up studies.

### Statistical Analyses

Statistical analyses were performed using the Statistical Package for the Social Sciences (SPSS version 20, USA). The demographic data were calculated using descriptive statistics. The categorical data were presented as frequencies and percentages (%). The numerical data were reported as the mean and standard deviation (SD) or the median and quartile. The chi-squared test for categorical variables, such as gender, Was taken into account. All continuous variables with normal distribution were calculated by two independent-sample *T*-test between schizophrenia and HC groups, while the Mann-Whitney *U*-test was used when the distribution was not normal. Partial correlation analysis was adopted to examine the relationship between leptin methylation, mRNA expression and psychopathology of schizophrenia, with the following covariates when appropriate: gender, age, BMI, dose of antipsychotics and duration of illness. Taking the psychopathology scores as dependent variables and gender, age, BMI, dose of antipsychotic, duration of illness, LEP-CpG methylation and LEP-mRNA expression as independent variables, we used multiple linear stepwise regression analysis to explore potential factors influencing psychopathology symptoms. The significant statistical level was set at *P*-value < 0.05.

## Results

### Demographic Data of Patients With Schizophrenia and HC

All analyses were performed on 40 patients and 40 controls. There were no significant differences in age, gender or BMI between the HC and schizophrenic patients (as shown in Table 1). Only significant differences in education were found when comparing patients and controls (*P* < 0.05).

### Leptin CpG Site Methylation and mRNA Expression

**Figure 2:**
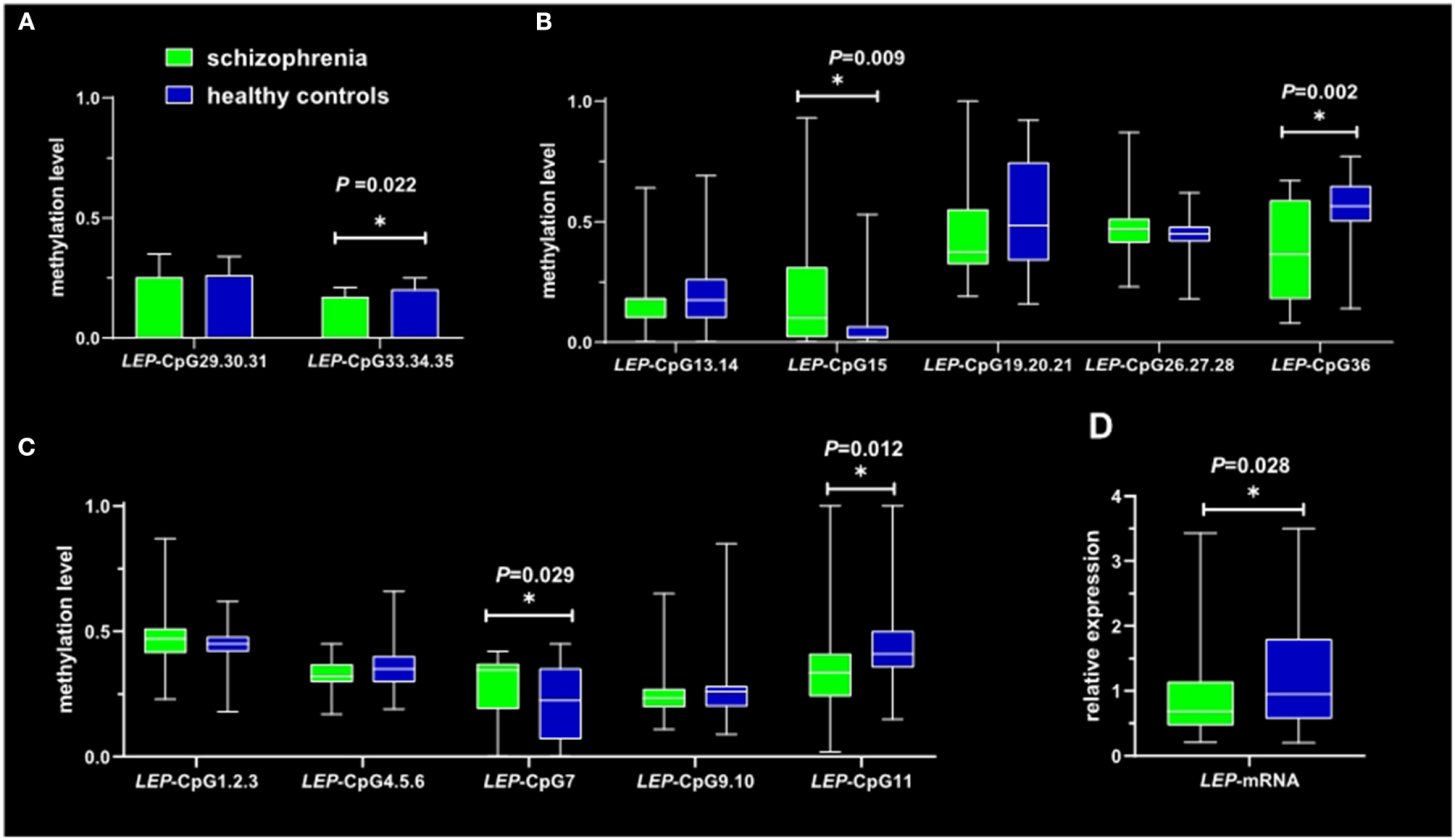
Between-group comparison of Leptin CpG site methylation and mRNA expression. The two-sample *t*-test was used to assess the differences in leptin CpG sites methylation **(A)** in the promoter resign between patients and healthy groups. A Mann-Whitney *U*-test **(B–D)** was used to assess the differences in leptin CpG sites methylation which were consisted with abnormal distribution. ^*^P < 0.05.

Although significant differences existed in education between patients and HC groups, it was not involved in analyzing covariates due to absence of correlation between education and leptin methylation (*P* > 0.05). *LEP*-CpG7 (*z* = −2.182, *P* = 0.029) and *LEP*-CpG15 (*z* = −2.622, *P* = 0.009) methylation surrounding the leptin promoter region in schizophrenic inpatients were significantly higher than those in HC. In contrast, markedly lower DNA methylation levels were observed in *LEP*-CpG11 (*z* = −2.517, *P* = 0.012), *LEP*-CpG33.34.35 (*t* = −2.342, *P* = 0.022) and *LEP*-CpG36 (*z* = −3.096, *P* = 0.002) methylation in patients. In addition, patients with schizophrenia exhibited significant lower *LEP*-mRNA expression (*z* = −2.203, *P* = 0.028) when compared with HC groups after using the Mann-Whitney *U*-test (Figure 2). There was no significant difference in other leptin CpG sites methylation between patients and HC groups (*P* > 0.05) (Figure 2) (details see Supplementary Table 1).

### Correlation Between Leptin CpG Methylation, mRNA Expression, and Pathophysiology in Schizophrenic Patients

After controlling gender, age, BMI, dose of antipsychotic and duration of illness, partial correlation analysis showed a negative correlation between *LEP*-CpG7 methylation and PANSS positive subscore (*r* = −0.485, *P* = 0.005). In addition, *LEP*-mRNA expression demonstrated a negative correlation with PANSS total score (*r* = −0.385, *P* = 0.03) and positive subscale (*r* = −0.392, *P* = 0.026; Table 2). With the exception of *LEP*-CpG7 and *LEP*-mRNA, there was no significant correlation between other leptin CpG sites methylation and PANSS scores as well as depression scores in patients with schizophrenia (*P* > 0.05).

**Table 2:** Partial correlations between *LEP*-CpG methylation, *LEP*-mRNA expression and PANSS scores in patients with schizophrenia, controlled for gender, age, BMI, dose of antipsychotic, and duration of illness (*n* = 40).

	***LEP*** **-CpG7**		***LEP-*** **mRNA**	
	***r***	***P-*values**	***r***	***P-*values**
PANSS total score	−0.305	0.089	−0.385	0.030^*^
Positive subscale	−0.485	0.005^*^	−0.392	0.026^*^
Negative subscale	−0.102	0.579	−0.243	0.180
General psychopathology subscale	−0.212	0.244	−0.324	0.070
Depressed subscale	−0.036	0.846	−0.243	0.180

*PANSS, Positive and Negative Syndrome Scale. Depressed subscale was the total score of G2, G3 and G6 from the General psychopathology subscale*.

* *P < 0.05*.

### Regression Analysis of Psychopathology in Schizophrenia Patients

**Figure 3:**
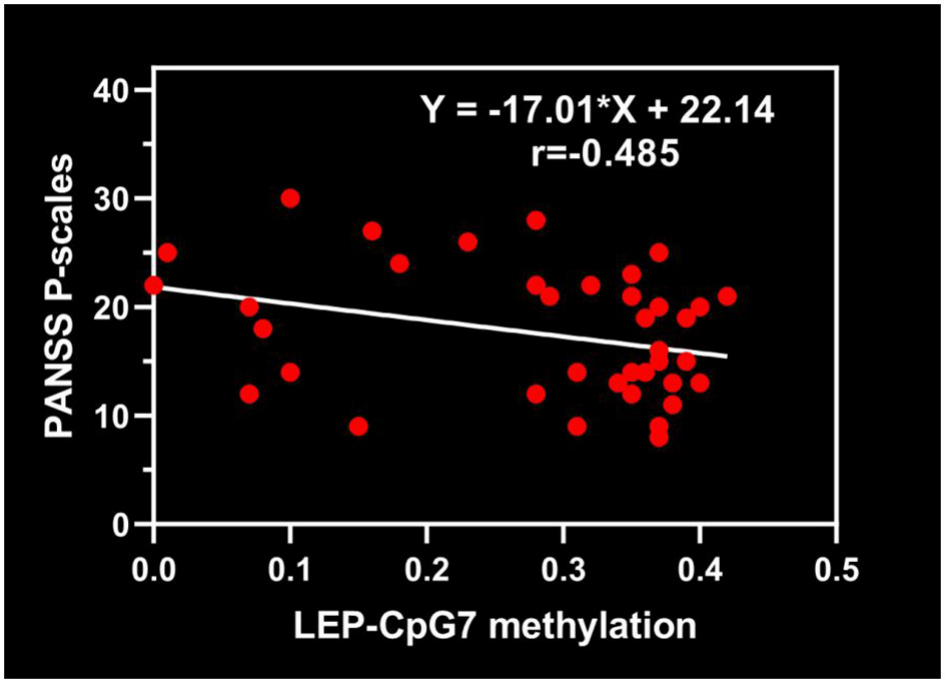
Multiple stepwise regression between *LEP*-CpG7 methylation and PANSS Positive subscore.

Furthermore, multiple linear stepwise regression was used to predict the psychopathology with variables, which are shown to have a significant correlation with PANSS score in Table 2. Following exclusion of *LEP*-mRNA expression, the *LEP*-CpG7 methylation level remained negatively correlated to PANSS positive subscore, with correlation coefficients of −0.485 (β = −17.071, *t* = −2.170, *P* = 0.037). Meanwhile, *LEP-*mRNA expression was not significantly correlated with PANSS total score due to being excluded from the linear regression (β = −5.951, *t* = −1.601, *P* = 0.118; Figure 3).

## Discussion

As far as we know, this is the first study to investigate leptin CpG sites methylation in the promoter region and mRNA expression changes using the MassARRAY system and RT-PCR in patients with schizophrenia. We further explored the relationship between leptin methylation, mRNA expression and clinical symptoms in PANSS. Here, we presume that schizophrenic patients may undergo abnormal DNA promoter methylation and mRNA expression of leptin, an important neurodevelopmental gene, and it probably plays an important role in pathogenesis of schizophrenia. Furthermore, LEP-CpG7 methylation may be negatively associated with positive symptoms in patients with schizophrenia.

In this study, we observed significantly higher *LEP*-CpG7, *LEP*-CpG15 methylation and lower *LEP*-CpG11, *LEP*-CpG33.34.35, *LEP*-CpG36 methylation in inpatients with schizophrenia, which were never reported in previous studies. There was significant up-regulated leptin mRNA expression in schizophrenia compared with HC subjects. To our knowledge, no previous human study targeted pathogenesis as well as psychotic symptom changes in schizophrenia in relation to DNA methylation and mRNA expression within the leptin gene. In view of the limited power, the significant association of leptin methylation, mRNA expression and schizophrenia is noteworthy. Early research in schizophrenic patients paid main attention to the relationship between serum or plasma leptin levels and weight gain due to typical or atypical antipsychotic treatment (29, 30). Several studies demonstrated that increased leptin levels were heavily dependent on weight gain or BMI in patients with schizophrenia (24). However, a recent finding shown that schizophrenia was still closely associated with increased leptin levels compared with the healthy when considering gender, age and BMI (31). Our present finding of elevated leptin mRNA expression in patients with schizophrenia supports this speculation. As we all know, leptin mRNA is translated into functional protein, which has essential effect in the peripheral blood and other tissues (32). However, our study did not find a certain relationship between methylation and mRNA expression, which may be disturbed by other factors, such as acetylation, histone modification, and non-coding RNA regulation, during transcription (33). So more research is needed to explore internal specific and profound mechanisms in the future.

Altogether, the recent evidence suggests that leptin, which induced potential hippocampal synaptic neuroplasticity (34), may be play a great role in the pathophysiology of schizophrenia. However, recent results of leptin in schizophrenic patients were conflicting. Some research showed that leptin levels increased, not only in patients undergoing atypical antipsychotics (35) but also in drug-free (36) and drug-naïve schizophrenic patients (37). In the past, most studies were performed on serum protein, and a few researchers have explored the underlying specific hereditary mechanism of the leptin dysregulation hypothesis. There is little information on polymorphism of leptin genes in schizophrenic patients. German researchers found that the G allele of the−2548A/G (*LEP*: rs7799039) polymorphism in the promoter neighborhood of *LEP* was not associated with significantly greater olanzapine- and clozapine-induced weight gain (38). Klemettil's findings also supported the similar negative conclusion (39). However, the inconsistent results regarding leptin between schizophrenia and healthy subjects could not be explained by the polymorphism of the leptin gene in its entirety. Hence, increasing research indicated that differential DNA methylation and mRNA expression was shown to be associated with schizophrenia (40), affecting neuronal cell size, dendritic growth and synaptic density, which are all implicated in schizophrenia development (41). Additionally several previous studies shown that central nervous gene expression could be replaced by that in peripheral blood leukocytes based on their considerable gene expression similarities (42, 43). Based on the above, we carry out a trial to explore the role of leptin CpG sites methylation and mRNA expression in peripheral blood of schizophrenic patients. For the first time, we put forward a hypothesis of abnormal regulation of leptin methylation and mRNA expression in patients with schizophrenia. Although it is needed that further studies will be carried out for confirmation. To some extent, these results further confirm that schizophrenia is related to abnormal regulation of multiple genes, and has important reference value for future research on the pathogenesis of this disease.

Recently, these emerging studies have focused on potential links between leptin dysregulation and clinical psychopathology (44). In the current study, there was also a significant correlation between the abnormal leptin methylation, mRNA expression and schizophrenic symptoms. Potvin et al. recognized that higher serum leptin levels were associated with less severe positive symptoms of schizophrenia (30). Nevertheless, Gamze Erzin et al. illustrated that there was no significant correlation between the terms of positive, negative, cognitive subscales and leptin levels (45). Some inconsistencies are presented in literature regarding the differential link between clinical symptoms of schizophrenia and leptin in peripheral blood. We think that the inconsistent results of studies may be due to the lack of consideration of mixed factors, such as antipsychotics, course of disease, gender, BMI, etc. Jow et al. indicated that higher leptin levels were observed in schizophrenic patients without antipsychotic drugs (22). This study did not control body weight in the analysis, which might be the reason for higher leptin levels in patients compared to HC groups. Meanwhile, some researchers (46) proposed that the duration of illness in patients had to be taken into account when analyzing data. In summary, taking gender, age, BMI, dose of antipsychotics and duration of illness into consideration, we observed a negative correlation between *LEP*-CpG7 methylation and PANSS positive subscales in patients. Moreover, *LEP*-mRNA expression exhibited a negative link with PANSS total score and positive subscales. Regression analysis suggested that only *LEP*-CpG7 methylation was negatively associated with positive symptoms in patients with schizophrenia. We speculated that there was only a simple correlation between mRNA and symptoms in schizophrenia patients under the susceptible influence of various factors, but on a whole, without predicting the severity of disease. So this potential link about psychopathological symptoms and epigenetic mechanism need more lager samples and longitudinal tests to be further confirmed.

Furthermore, given the complexity of the symptoms of schizophrenia and previous results, which shown that declined leptin occurred in patients with severe suicidal behavior (47) or depression (48), we listed and analyzed the depressive factors solely. However, the result is not satisfactory when compared to previous studies. We assumed that previous analysis might not control confounding variables, such as dose of antipsychotics, duration of illness etc., or that abnormal leptin expression might be not specific for depressive symptoms of schizophrenia. It is hoped that further experimentation in this regard will be carried out for confirmation.

## Limitations

Several limitations of this study are noted. Firstly, the cross-sectional study does not induce us to draw conclusions regarding the orientation of the association between leptin CpG site methylation, mRNA expression and schizophrenic patients. Secondly, as the sample size is small and leptin gene expression is susceptible to multiple factors, our findings should be deemed to preliminary and profound exploration. And subsequent research needs to strictly calculate the sample size. Thirdly we didn't adjust for multiple comparisons in the correlation analysis. So these maybe increase the possibility of type 1 error. Lastly, although we tried our best to control dose of antipsychotics as covariates during data analysis, we could not avoid that antipsychotics themselves have a certain degree of influence on DNA methylation and mRNA expression. As several researches provided more information on the impact of antipsychotics on leptin levels than the association between schizophrenia and leptin (46). Results that are more meaningful will be acquired if future studies consider using first-episode drug-naïve (FEDN) individuals with schizophrenia.

## Conclusion

In conclusion, this study indicated that there was leptin CpG site methylation in the promoter region and mRNA expression dysregulation in schizophrenia inpatients. These results examine the possibility that the leptin gene acts as a schizophrenia susceptibility gene owing to its combination of both genetic and epigenetic transcriptional gene regulation. *LEP*-CpG7 methylation was negatively associated with positive symptoms in patients with schizophrenia. The hypothesis of DNA methylation and mRNA expression is noteworthy to identify a new way to explore the pathogenesis of schizophrenia.

## Data Availability Statement

The raw data supporting the conclusions of this article will be made available by the authors, without undue reservation.

## Ethics Statement

The studies involving human participants were reviewed and approved by the Ethics Committee of Beijing Huilongguan Hospital. The patients/participants provided their written informed consent to participate in this study. Written informed consent was obtained from the individual(s) for the publication of any potentially identifiable images or data included in this article.

## Author Contributions

JS, YC, DC, and WL wrote and revised the manuscript. JS, KC, and HL analyzed the data. QZ, JY, and WF contributed to data collection and management. DC provided essential suggestions and revised the manuscript. All authors contributed to and have approved the final manuscript.

## Funding

This study was supported by the Capital Health Research and Development of Special (Grant: SF2018-2-2131).

## Conflict of Interest

The authors declare that the research was conducted in the absence of any commercial or financial relationships that could be construed as a potential conflict of interest.

## Publisher's Note

All claims expressed in this article are solely those of the authors and do not necessarily represent those of their affiliated organizations, or those of the publisher, the editors and the reviewers. Any product that may be evaluated in this article, or claim that may be made by its manufacturer, is not guaranteed or endorsed by the publisher.
