# Supplementary material for: Leptin Methylation and mRNA Expression Associated With Psychopathology in Schizophrenia Inpatients
Source: Front Psychiatry. 2022 Feb 7;13:793910. doi: 10.3389/fpsyt.2022.793910 (PMC8858839; doi:10.3389/fpsyt.2022.793910)
Supplement: Supplementary file 1 [file Table_1.DOC]

Spplementary table 1. Differences in the *LEP*-CpG methylation level and *LEP*-mRNA between schizophrenia patients and healthy controls

| CpG site | Schizophrenia(n=40) | Healthy controls(n=40) | *t or Z* | *P* values |
| --- | --- | --- | --- | --- |
| *LEP*-CpG1.2.3 | 0.47（0.42,0.51） | 0.45（0.42,0.48） | -1.121 | 0.262 |
| *LEP*-CpG4.5.6 | 0.32（0.30,0.37） | 0.35（0.30,0.40） | -1.158 | 0.247 |
| *LEP*-CpG7 | 0.35（0.19,0.37） | 0.23（0.07,0.35） | -2.182 | 0.029^*^ |
| *LEP*-CpG9.10 | 0.24（0.20,0.27） | 0.26（0.20,0.28） | -1.008 | 0.314 |
| *LEP*-CpG11 | 0.34（0.24,0.41） | 0.41（0.36,0.50） | -2.517 | 0.012^*^ |
| *LEP*-CpG13.14 | 0.18（0.10,0.19） | 0.18（0.10,0.26） | -0.728 | 0.467 |
| *LEP*-CpG15 | 0.10（0.02,0.31） | 0.02（0.02,0.07） | -2.622 | 0.009^*^ |
| *LEP*-CpG19.20.21 | 0.38（0.32,0.55） | 0.49（0.34,0.75） | -1.247 | 0.212 |
| *LEP*-CpG26.27.28 | 0.47（0.42,0.51） | 0.45（0.42,0.48） | -1.628 | 0.103 |
| *LEP*-CpG29.30.31 | 0.25±0.10 | 0.26±0.08 | 0.482 | 0.631 |
| *LEP*-CpG33.34.35 | 0.17±0.04 | 0.20±0.05 | 2.342 | 0.022^*^ |
| *LEP*-CpG36 | 0.37（0.18,0.59） | 0.57（0.50,0.65） | -3.096 | 0.002^*^ |
| *LEP*-mRNA | 0.69（0.48,1.14） | 0.95（0.58,1.79） | -2.203 | 0.028^*^ |

Abbreviations: All variables are presented as mean ± SD (accordance with normal distribution: A) or M (P25, P75). * *P* < 0.05
