# Supplementary figures and images for: Leptin Methylation and mRNA Expression Associated With Psychopathology in Schizophrenia Inpatients
Source: Front Psychiatry. 2022 Feb 7;13:793910. doi: 10.3389/fpsyt.2022.793910 (PMC8858839; doi:10.3389/fpsyt.2022.793910)

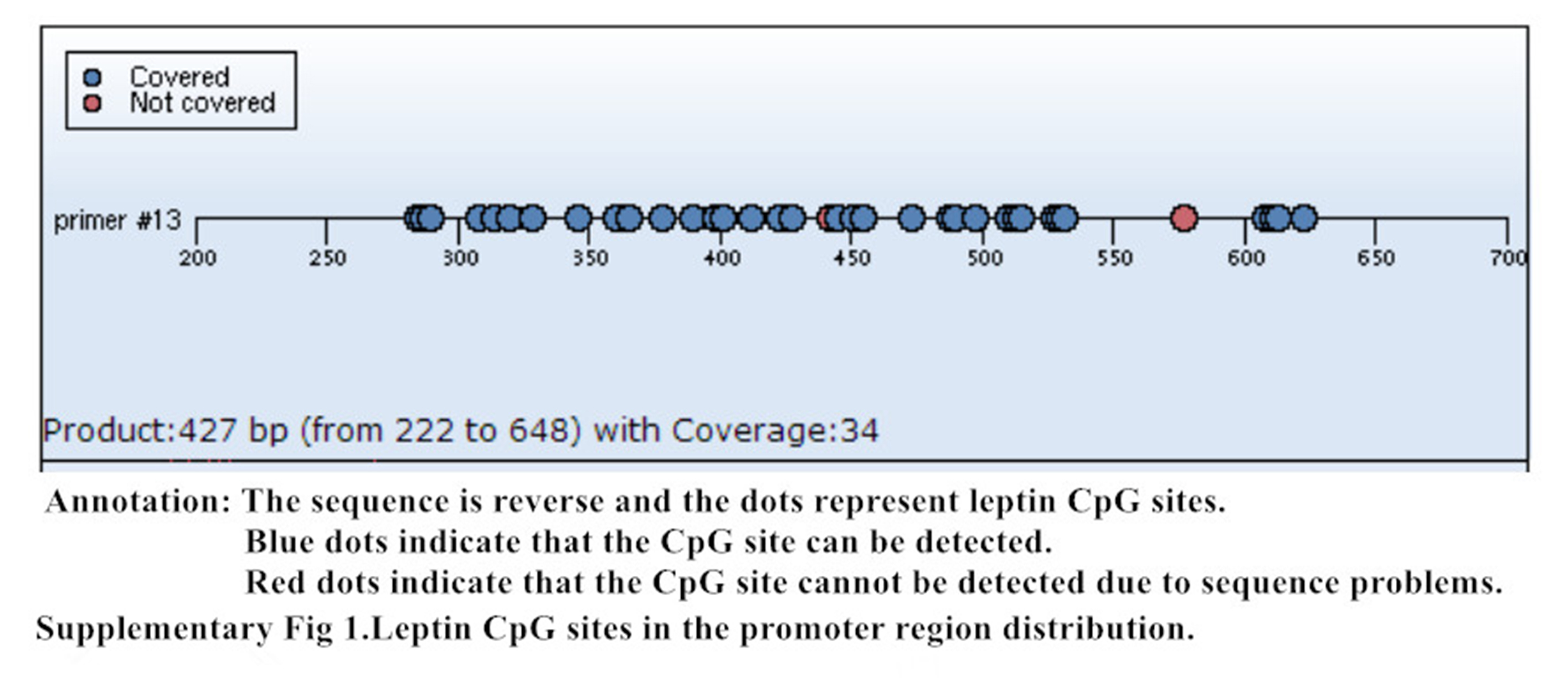

Supplement: Supplementary file 2 [file Image_1.JPEG]
